# Transcriptome profiling provides insights into leaf color changes in two *Acer palmatum* genotypes

**DOI:** 10.1186/s12870-022-03979-x

**Published:** 2022-12-16

**Authors:** Lu Zhu, Jing Wen, Qiuyue Ma, Kunyuan Yan, Yiming Du, Zhu Chen, Xiaoyu Lu, Jie Ren, Yuelan Wang, Shushun Li, Qianzhong Li

**Affiliations:** 1 Institute of Leisure Agriculture, Jiangsu Academy of Agricultural Sciences, 50 Zhongling Street, 210014 Nanjing, Jiangsu China; 2 Institute of Agricultural Engineering, Anhui Academy of Agricultural Sciences, 40 Nongke South Road, 230031 Hefei, Anhui China; 3 Chenshi Maples Nursery, 313308 Longba Village, Huzhou, Zhejiang China

**Keywords:** *Acer palmatum*, Transcriptome, Leaf coloration, Anthocyanin, Transcription factors

## Abstract

**Background:**

Ornamental trees with seasonally-dependent leaf color, such as *Acer palmatum*, have gained worldwide popularity. Leaf color is a main determinant of the ornamental and economic value of *A. palmatum*. However, the molecular mechanisms responsible for leaf color changes remain unclear.

**Results:**

We chose *A. palmatum* cultivars with yellow (‘Jinling Huangfeng’) and red (‘Jinling Danfeng’) leaves as the ideal material for studying the complex metabolic networks responsible for variations in leaf coloration. The 24 libraries obtained from four different time points in the growth of ‘Jinling Huangfeng’ and ‘Jinling Danfeng’ was subjected to Illumina high-throughput sequencing. We observed that the difference in cyanidin and delphinidin content is the primary reason behind the varying coloration of the leaves. Transcriptomic analyses revealed 225,684 unigenes, and the Kyoto Encyclopedia of Genes and Genomes (KEGG) enrichment analysis of differentially expressed genes (DEGs) confirmed that they were involved in ‘anthocyanin biosynthesis.’ Eighteen structural genes involved in anthocyanin biosynthesis were thought to be related to anthocyanin accumulation, whereas 46 MYBs, 33 basic helix-loop-helixs (bHLHs), and 29 WD40s were presumed to be involved in regulating anthocyanin biosynthesis. Based on weighted gene co-expression network analysis (WGCNA), three candidate genes (*ApRHOMBOID*, *ApMAPK*, and *ApUNE10*) were screened in the significant association module with a correlation coefficient (*r*^*2*^) of 0.86.

**Conclusion:**

In this study, the leaf color changes of two *A. palmatum* genotypes were analyzed. These findings provide novel insights into variations in leaf coloration and suggest pathways for targeted genetic improvements in *A. palmatum*.

**Supplementary Information:**

The online version contains supplementary material available at 10.1186/s12870-022-03979-x.

## Background

Maple trees from the genus *Acer* (Aceraceae) are highly attractive species used for landscape decoration. They have high ornamental value due to their seasonally changing leaf color. Worldwide, there are over 200 species in the genus *Acer*, mainly distributed in the temperate regions of Asia, Europe, and America [1]. Of these, *A. palmatum* is the most frequently cultivated horticultural variety, being widely used in landscaping. *A. palmatum* has an aesthetically pleasing appearance, a distinctive leaf shape, and exhibits stunning diverse seasonal changes in leaf color [2].

The seasonal changes in leaf coloration are one of the most sought-after traits of *A. palmatum*. The mechanism responsible for leaf coloration changes is highly complex and is determined by the metabolite composition of the leaves. During the seasonal changes in leaf coloration, the leaves undergo numerous biochemical changes, including changes in the content and distribution of chlorophyll, carotenoids, and anthocyanins [3, 4]. Anthocyanins are a major contributor to non-green coloration in leaves [5, 6]. These bioactive compounds are flavonoids, which provide a wide variety of colors to various tissues, including the leaves, flowers, fruit, seeds, and roots, and are responsible for a red, orange, or blue-violet appearance [7, 8]. About 20 base anthocyanidins and more than 600 anthocyanins have been reported [9], with the most common anthocyanins being pelargonidin, cyanidin, delphinidin, peonidin, petunidin, and malvidin and their derivatives [10, 11]. An important branch of flavonoid synthesis, anthocyanin synthesis is relatively conserved in higher plants, and has been the subject of detailed research [4, 12]. Anthocyanin biosynthesis can be divided into three stages, starting from phenylalanine as the initial substrate for synthesis. In the first stage, phenylalanine ammonia-lyase (PAL), cinnamic acid-4-hydroxylase (C4H), and 4-coumaric acid coenzyme A ligase (4CL) catalyze the conversion of phenylalanine to 4-coumaroyl-CoA. In the second stage, 4-coumaroyl-CoA and malonyl-CoA are catalyzed by chalcone synthase (CHS), chalcone isomerase (CHI), flavanone-3-hydroxyl enzyme (F3H), flavonoid 3′-hydroxylase (F3′H), and flavonoid 3′5′-hydroxylase (F3′5′H) in order to generate dihydroflavonol. The third stage involves the formation of various anthocyanins. Dihydroflavonol 4-reductase (DFR) and anthocyanin synthase (ANS) catalyze the synthesis of anthocyanins from dihydroflavonol. The anthocyanins are further modified by a series of glycosylation steps under the action of flavonoid glucosyltransferase (UFGT) and methyltransferase (MT), in which the compounds combine with UDP-glucose to form stable anthocyanins [7, 10, 13, 14].

Synthesis, transport, and accumulation of anthocyanins are specifically and cooperatively regulated by various transcription factors. The most critical transcription factors in the anthocyanin synthesis pathway are thought to be MYB, bHLH, and WD40 [15, 16]. Three families of transcription factors can promote or inhibit anthocyanin biosynthesis by recognizing and binding specific regions of structural gene promoters [17–19] or by combining to form the MYB-bHLH-WD40 (MBW) complex which jointly regulates transcription of synthesis genes [15, 20]. The MBW complex plays a key role in promoting or inhibiting anthocyanin metabolism, and this regulatory mode has been confirmed in a variety of plants such as morning glory, apple, chrysanthemum, and *Freesia hybrid* [15, 17–19]. The MYB transcription factor is a critical element in the MBW complex. The MYB activators of anthocyanin biosynthesis are mainly from the R2R3-MYB sub-family, and MYB repressors consist of both the R2R3-MYB and R3-MYB subfamilies [21, 22]. Compared to the MYB transcription factor regulating individual genes acting in anthocyanin synthesis, the MBW complex more efficiently and coordinately regulates genes related to synthesis, modification, and transport in the anthocyanin synthesis pathway [23]. The MBW complex mainly relies on bHLHs, which recognize specific binding sites in target gene promoters and activate transcription [24]. As a crucial member of the MBW complex, WD40 proteins promote protein interactions and provide a stable platform for MYB and bHLH to form transcription complexes [25].

RNA-sequencing (RNA-seq), based on transcriptome profiling, has become an efficient tool for identifying gene expression patterns in different environments beyond model organisms and without reference sequences. Transcriptome data is effective in elucidating the molecular mechanisms responsible for plant growth and development and enables the identification of genes valuable to further research [26]. RNA-seq has been widely used in plants and has provided new insights into the evolution of plant sequences.

Although the anthocyanin biosynthesis pathway and the MBW complex have been extensively studied, we still lack a complete picture of the molecular mechanisms responsible for anthocyanins and their effects on leaf color in *A. palmatum*. In this study, we performed physicochemical and transcriptome analyses in two cultivars of *A. palmatum*, ‘Jinling Danfeng’ and ‘Jinling Huangfeng’. ‘Jinling Danfeng’ is a new variety with stable inherited red leaves that was found after tissue culture of ‘Jinling Huangfeng’, which has yellow leaves. Our goal was to identify related genes and transcription factors that could then be used to monitor the pigment changes in red and yellow leaves from spring to summer. The structural genes and transcription factors involved in anthocyanin biosynthesis were examined and three candidate genes were screened through weighted correlation network analysis (WGCNA). This study lays the foundation for further investigation into the mechanisms controlling leaf color in *A. palmatum*.

## Results

### Pigment changes of red and yellow leaves from spring to summer

**Fig. 1:**
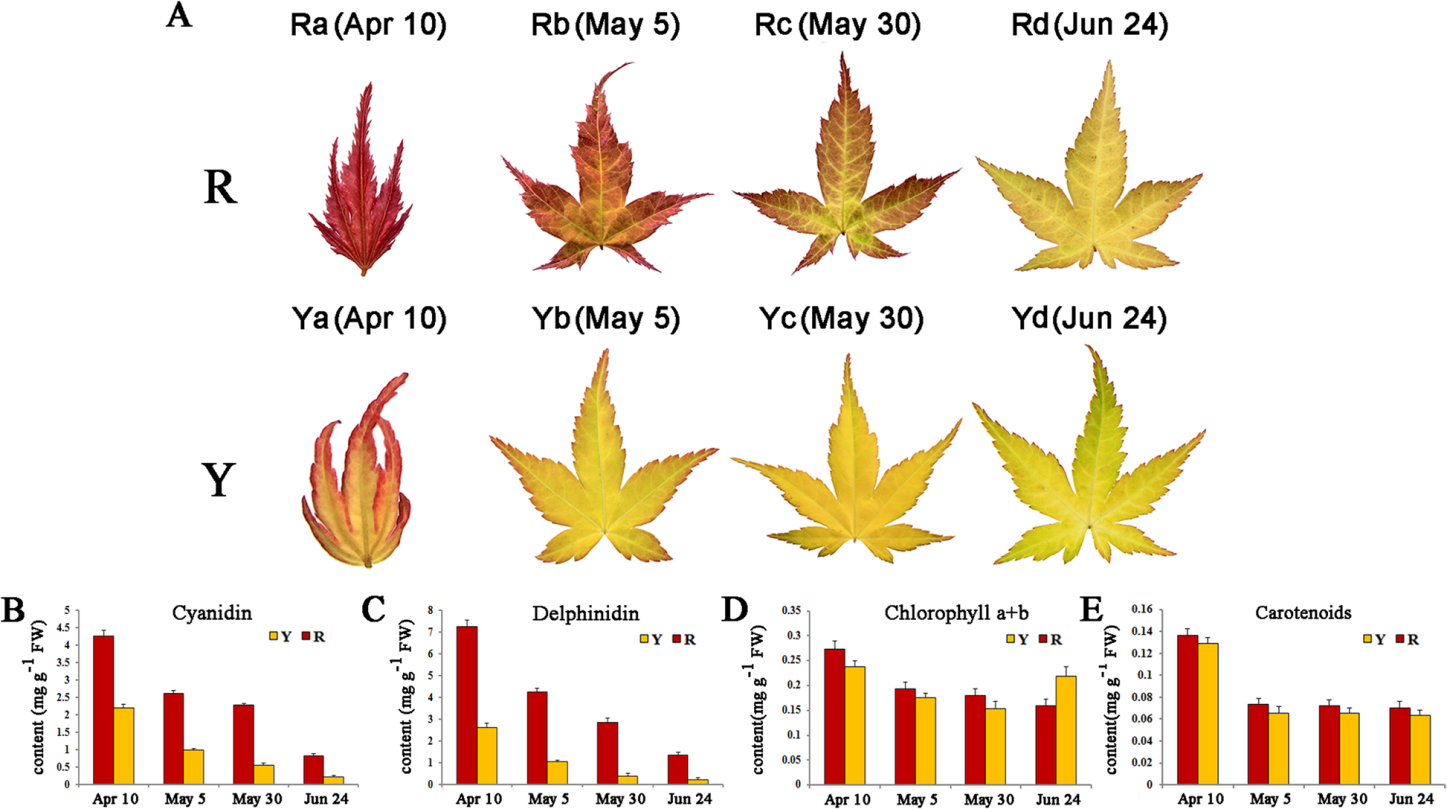
Morphology and pigment accumulation in two genotypes of *A. palmatum*. **A** Changes in the color of leaves of the red-leaf cultivar ‘Jinling Danfeng’ and yellow-leaf cultivar ‘Jinling Huangfeng’ from spring to summer. R: ‘Jinling Danfeng;’ Y: ‘Jinling Huangfeng.’ **B–E** Pigment content in different leaf colors of ‘Jinling Danfeng’ and ‘Jinling Huangfeng’: **B** Cyanidin; **C** Delphinidin; **D** Chlorophyll a + b; **E** Carotenes. The values derived from the data are represented as the mean ± SE (*n* = 3)

The young leaves of ‘Jinling Danfeng’ were initially bright red (Ra), and the color gradually turned light orange (Rb-Rd). The young leaves of ‘Jinling Huangfeng’ were initially yellow, and the leaf margins were light coral (Ya). The mature leaves gradually turned golden yellow (Yb, Yc) and then yellow-green (Yd) (Fig. 1A). To investigate the chemical basis of these phenotypic changes, the amounts of six anthocyanins, chlorophyll a + b, and carotenes in red and yellow leaves were determined and analyzed. Cyanidin and delphinidin were found to be major contributors to the color of red and yellow leaves, whereas pelargonidin, peonidin, petunidin, and malvidin were not detected in the leaves. The cyanidin and delphinidin content in the red-leaf cultivar ‘Jinling Danfeng’ was higher than that of the yellow-leaf cultivar ‘Jinling Huangfeng’ during the same period. However, the cyanidin and delphinidin content also gradually decreased as the redness of the leaves decreased (Fig. 1B, C). The chlorophyll a + b and carotenoid content registered minimal differences between the red- and yellow-leaf cultivars during the same period (Fig. 1D, E).

### Summary of transcriptome sequencing of *A. palmatum*

To elucidate the transcriptional mechanism of color changes in red and yellow leaves, RNA-seq experiments were performed on the red- and yellow-leaf cultivars. Twenty-four mRNA samples (Ra, Rb, Rc, Rd, Ya, Yb, Yc, and Yd, each in triplicate) were collected every 25 days from April 10 to June 24, and Illumina high-throughput sequencing was performed. Transcriptome sequencing generated 1,265,867,670 raw reads and 1,233,094,412 clean reads (97.41%, 184.97 Gb), with an average Q20 and Q30 values of 96.92% and 91.82%, respectively, and an average GC content of 43.91% (Table S1). After assembly, 225,684 non-redundant unigenes were identified. Among them, 169,533 (75.12%) of the unigenes ranged from 201 to 500 bp in length, 26,789 (11.87%) of the unigenes ranged from 501 to 1000 bp in length, 18,320 (8.12%) of the unigenes ranged from 1001 to 2000 bp in length, and 11,042 (4.89%) unigenes were > 2000 bp in length (Fig. S1).

### Functional annotation and classification of unigenes

**Fig. 2:**
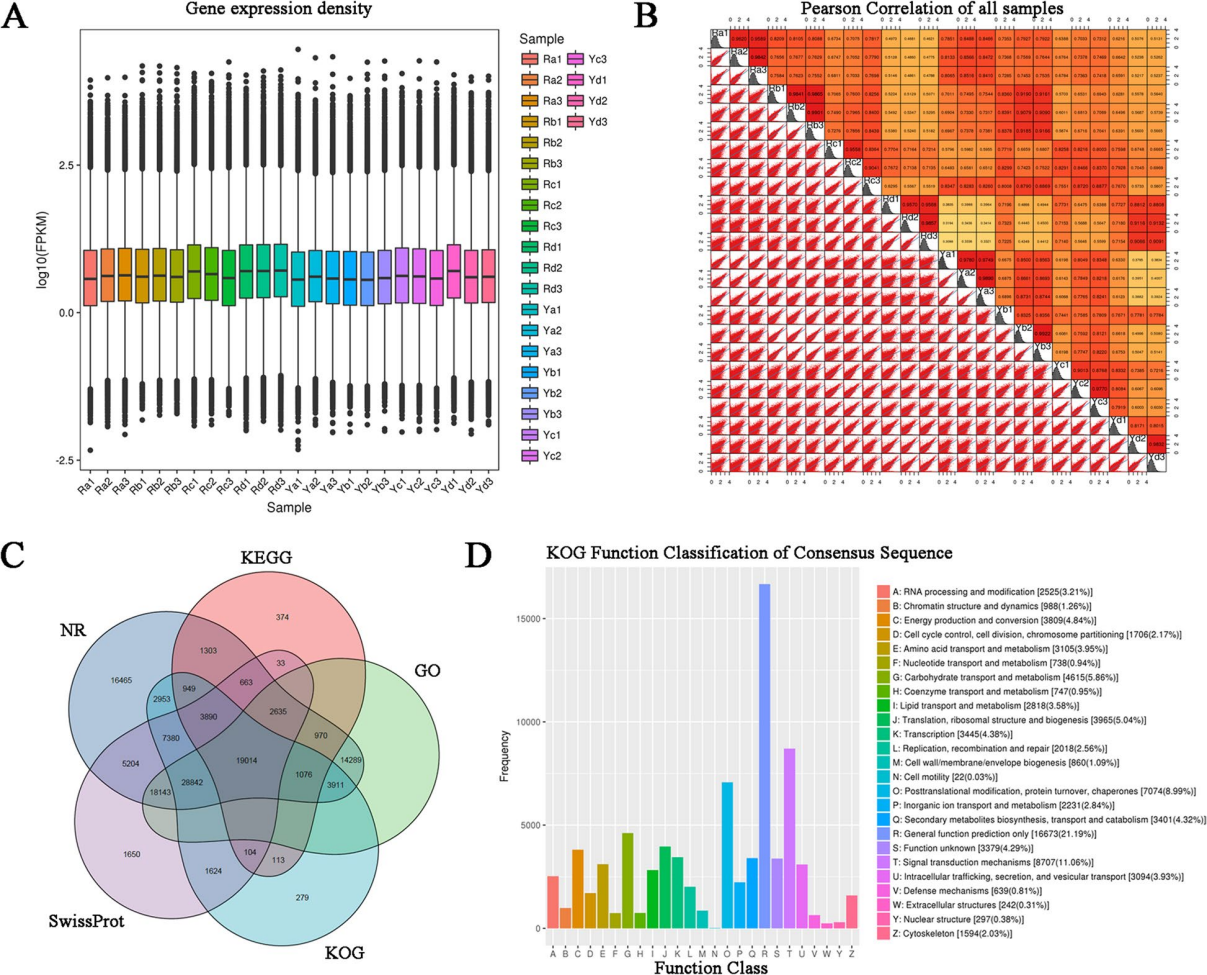
Overview of transcriptome sequencing results and annotation of unigenes. **A** Boxplot representing the distribution of expression levels of all the genes in each sample using their FPKM values. **B** Heat map for expression correlation between pairwise samples. **C** Venn diagram demonstrating the number of unigene annotations according to five databases. **D** KOG functional classification of all unigenes

Transcriptome expression profiling of all the samples revealed the genes that were expressed in spring to summer in the two *A. palmatum* genotypes. The expression levels of the genes varied among the different samples (Fig. 2A). A high Pearson coefficient was maintained between all replicates (*r* > 0.8), indicating a very high correlation in replicate samples (Fig. 2B). To determine the putative functions of the unigenes in *A. palmatum*, the assembled unigenes were functionally annotated against the NR, KEGG, GO, KOG, and SwissProt databases. A total of 131,864 unigenes were annotated in at least one database. A total of 19,014 (14.42%) unigenes were found to possess a homolog in these five databases. A total of 127,687 (96.83%), 31,124 (23.60%), 88,880 (67.40%), 70,135 (53.19%), and 89,182 (67.63%) unigenes were examined against the NR, KEGG, GO, KOG, and SwissProt databases, respectively (Fig. 2C). To further determine the primary biological functions of the unigenes, the KOG, GO, and KEGG pathways were analyzed. In total, 70,135 sequences were grouped into 26 KOG classifications. The top three categories were group R (general function prediction only 16,673, 21.19%), group T (signal transduction mechanisms 8,707, 11.06%), and group O (post-translational modification, protein turnover, chaperones 7,074, 8.99%) (Fig. 2D). GO analysis was used to describe the predicted unigenes. A total of 88,880 sequences were categorized into 51 functional groups, of which 15 belonged to cellular components, 17 to molecular functions, and 19 to biological processes (Fig. S2). A total of 31,124 unigenes were analyzed based on KEGG pathways, and these sequences were mapped to 19 KEGG pathway categories. The top three spots were occupied by ‘carbon metabolism’ (5383, 17.30%), ‘global and overview maps’ (3407, 10.95%), and ‘transduction’ (3323, 10.68%) (Fig. S3).

### Identification of DEGs

**Fig. 3:**
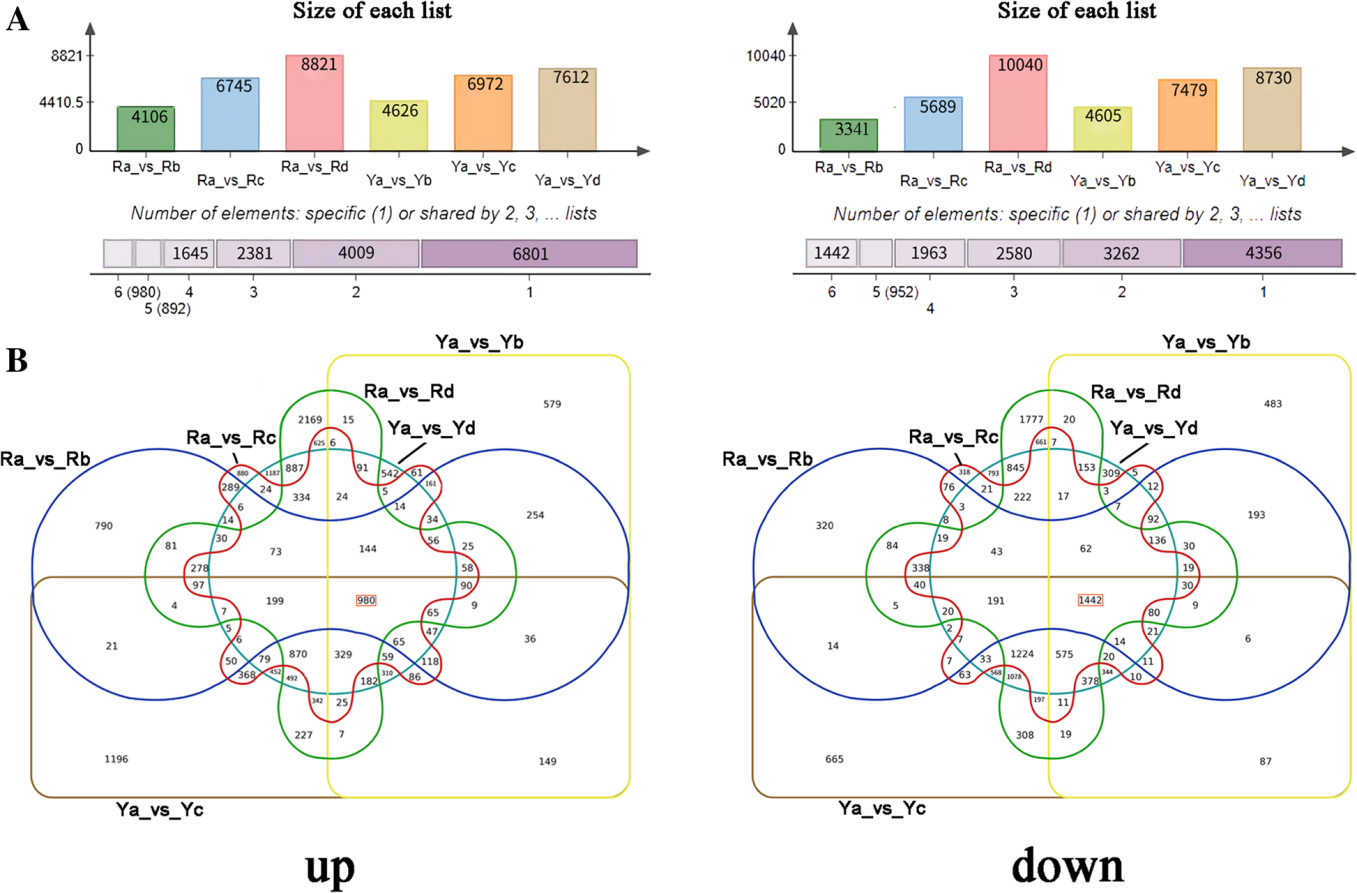
Unigenes transcribed differentially in two genotypes *A. palmatum*. **A** Summary of the number of up-regulated (left) and down-regulated (right) DEGs. **B** Venn diagram depicting the up-regulated (left) and down-regulated (right) genes between the trial groups

Significant differences in transcript abundance were identified in at least one pairwise comparison based on the FPKM values (Fig. 3, S4). Compared to other combinations, the Ra vs. Rd combination had the largest number of DEGs. A total of 8821 genes were up-regulated, and 10,040 genes were down-regulated. In contrast, the number of DEGs in the Ra vs. Rb combination was the lowest, where 4106 genes were up-regulated and 3341 genes were down-regulated (Fig. 3A). Notably, among the combinations Ya vs. Yb, Ya vs. Yc, and Ya vs. Yd, the two combinations with the most and least numbers of DEGs were Ya vs. Yd (7612 up-regulated and 8730 down-regulated) and Ya vs. Yb (4626 up-regulated and 4605 down-regulated), respectively (Fig. 3A). These results suggest that the number of DEGs gradually increased with the change in leaf color of *A. palmatum*. Further analysis indicated that 980 genes were commonly up-regulated in all six combinations, whereas 1442 genes were commonly down-regulated (Fig. 3B).

### KEGG analysis of DEGs

**Fig. 4:**
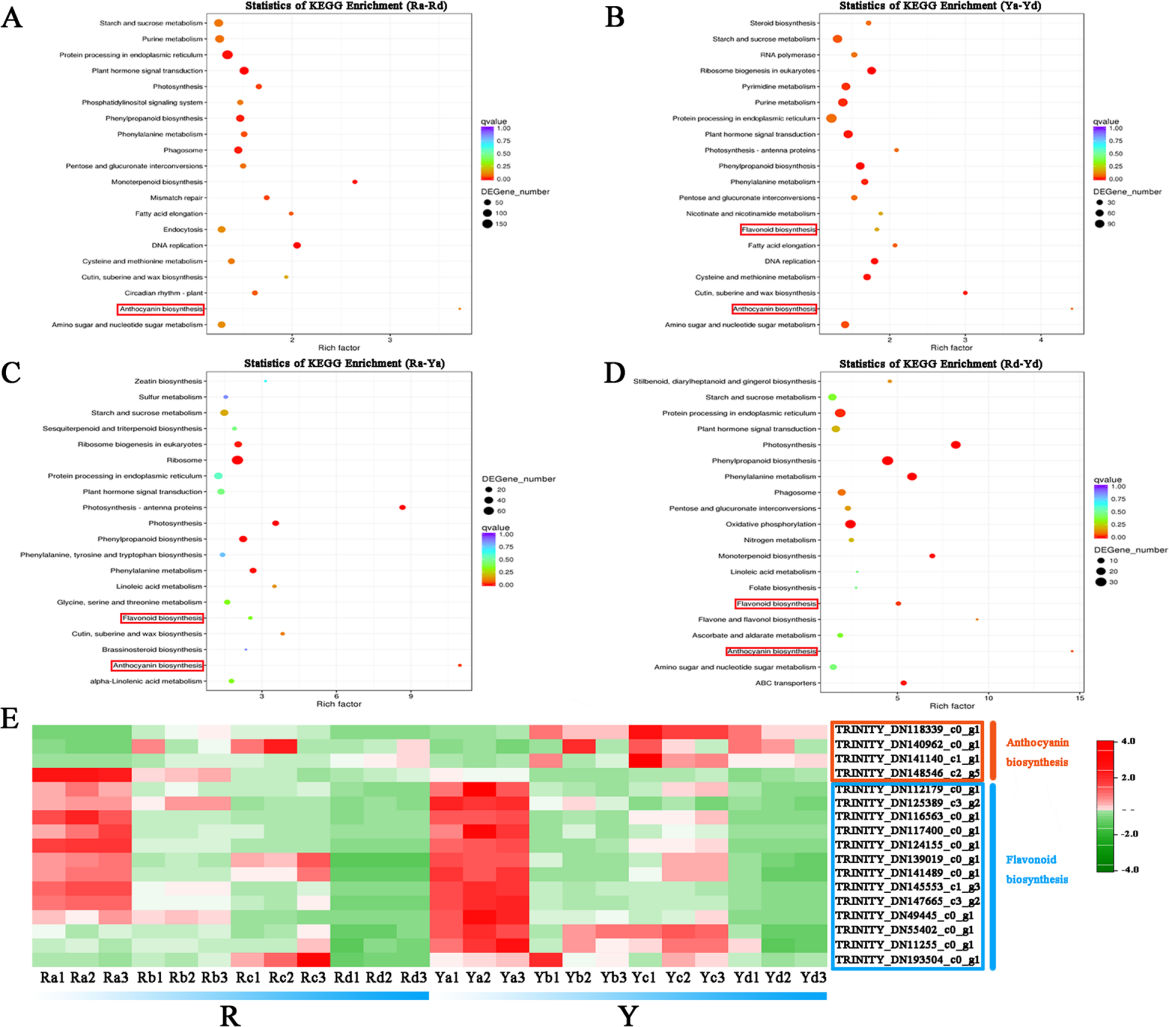
KEGG analysis of DEGs identified in samples Ra, Rd, Ya, and Yd (www.kegg.jp/kegg/kegg1.html). **A–D** Statistical results of DEGs KEGG enrichment. The red boxes represent key pathways involved in pigment accumulation. **A** Ra and Rd; **B** Ya and Yd; **C** Ra and Ya; **D** Rd and Yd. **E** Heat map constructed using the DEGs in the key pathways

All DEGs were queried in the KEGG pathway database, and four combinations (Ra vs. Rd, Ya vs. Yd, Ra vs. Ya, and Rd vs. Yb) were highlighted (Fig. 4A–D). The 20 most significantly enriched subcategories were displayed in the KEGG pathway enrichment. Two of the most prominent of these pathways were ‘anthocyanin biosynthesis’ and ‘Flavonoid biosynthesis,’ as these two pathways are closely related to the appearance of red and yellow coloring in the leaves of *A. palmatum*. For ‘anthocyanin biosynthesis,’ three of the four transcripts involved in this pathway had higher expression in Y compared to R. With regards to ‘flavonoid biosynthesis,’ 13 of the 15 transcripts involved in this pathway had the highest expression in Ra or Ya (Fig. 4E). These results highlight the involvement of anthocyanin and flavonoid biosynthesis in the red and yellow leaves of *A. palmatum*.

### Genes involved in anthocyanin biosynthesis

**Fig. 5:**
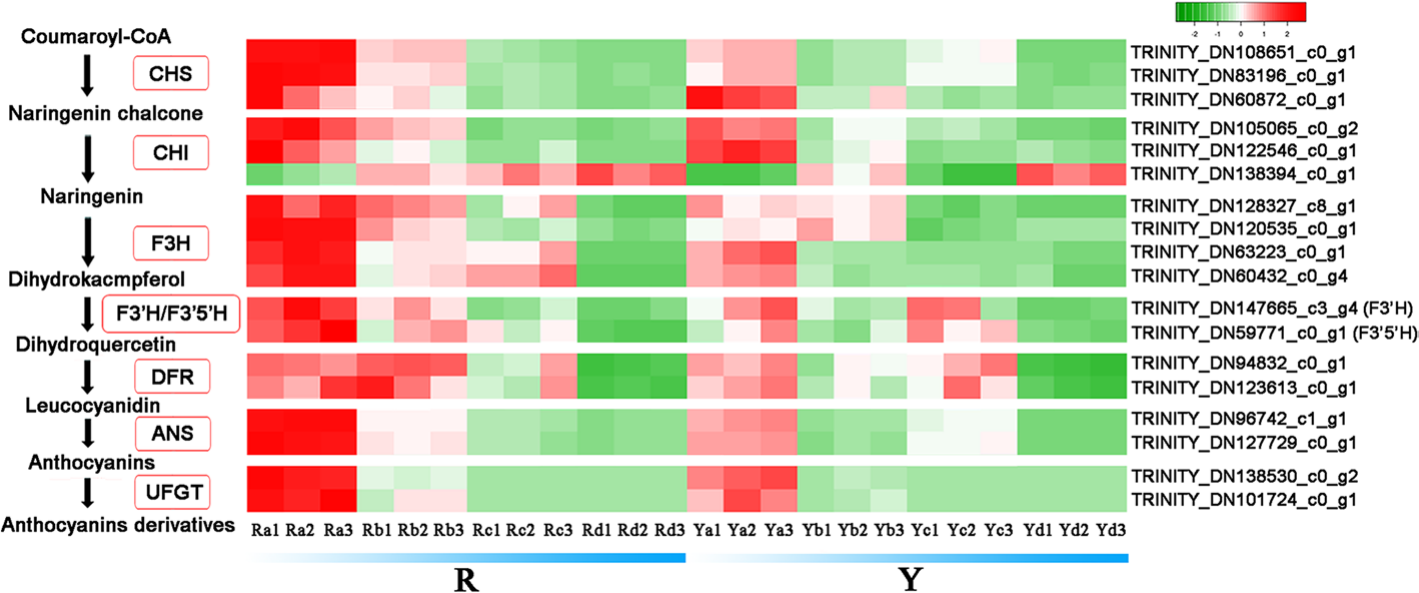
Heat map constructed using DEGs related to anthocyanin biosynthesis.; chalcone synthase (CHS), chalcone isomerase (CHI), flavanone 3-hydroxylase (F3H), flavanone 3′-hydroxylase (F3′H), flavanone 3′5′-hydroxylase (F3′5′H), dihydroflavonol 4-reductase (DFR), anthocyanin synthase (ANS) and anthocyanidin 3-O-glucosyltransferase (UFGT)

**Fig. 6:**
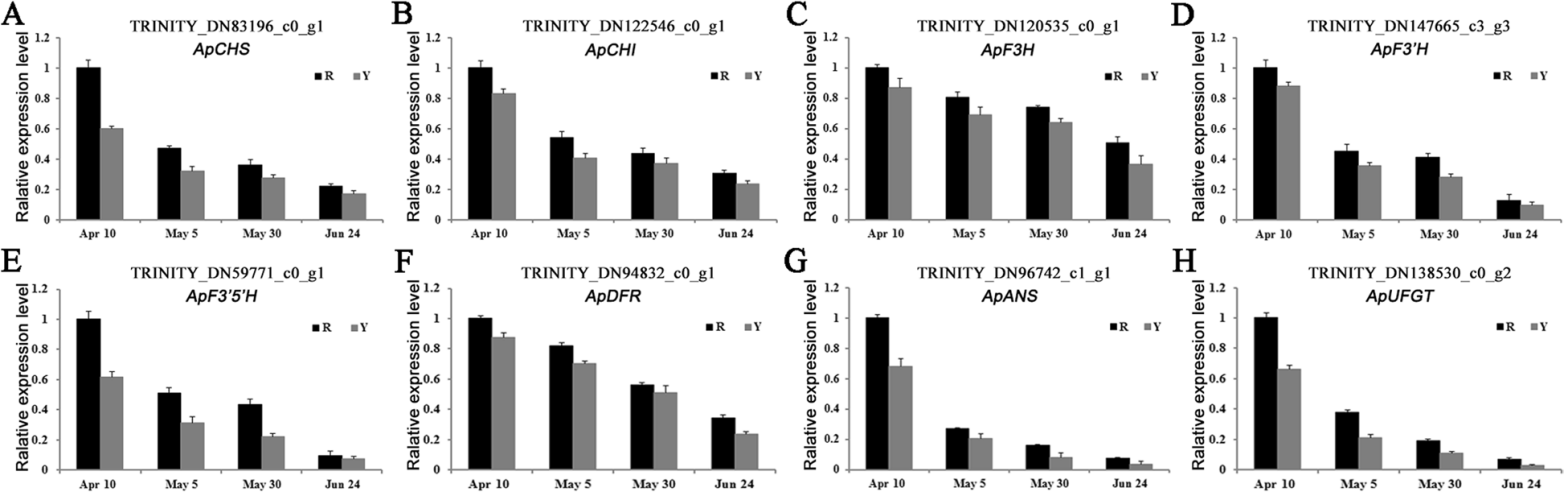
qRT-PCR verification of the expression pattern of key genes related to anthocyanin biosynthesis in two genotypes of *A. palmatum*. The values derived from the data are represented as the mean ± SE (n = 3). **A** *ApCHS*; **B** *ApCHI*; **C** *ApF3H*; **D** *ApF3′H*; **E** *ApF3′5′H*; **F** *ApDFR*; **G ** *ApANS*; **H ***ApUFGT*

Anthocyanin content is an important factor affecting leaf color, especially in red and yellow leaves. Accordingly, the key enzymes involved in the anthocyanin biosynthesis pathways were investigated. Eighteen candidate key genes, including *ApCHSs*, *ApCHIs*, *ApF3Hs*, *ApF3′Hs*, *ApDFRs*, *ApANSs*, and *ApUFGTs*, were mined from our transcriptome for further examination in our study, as these genes exert a direct influence over eight enzymes that are known to be involved in anthocyanin biosynthesis (Fig. 5). The expression level of all the members was investigated in detail within the two genotypes of *A. palmatum*. As shown in Fig. 5, most genes encoding enzymes regulated by the anthocyanin biosynthesis pathway demonstrated a similar expression pattern. Most transcripts exhibited the highest expression in Ra and the lowest in Rd and Yd. Only one gene exhibited a variation in expression pattern, consistent with it being a negative regulator of the anthocyanin biosynthesis pathway. The results indicated that consistency exists between the trends of anthocyanin accumulation and the expression pattern of key genes encoding enzymes involved in the anthocyanin biosynthesis pathway. In order to further characterize the expression of candidate genes linked to anthocyanin biosynthesis, seven DEGs, including *ApCHS*, *ApCHI*, *ApF3H*, *ApF3′H*, *ApDFR*, *ApANS*, and *ApUFGT*, were selected and identified as potential candidates for qRT-PCR experiments. Although the magnitude of the changes in candidate gene expression differed between qRT-PCR and RNA-seq, the trends observed in the expression patterns of these genes were consistent between the two methods, thereby validating the reliability of the RNA-seq results (Fig. 6).

## Identification of differential transcription factors related to anthocyanin biosynthesis

**Fig. 7:**
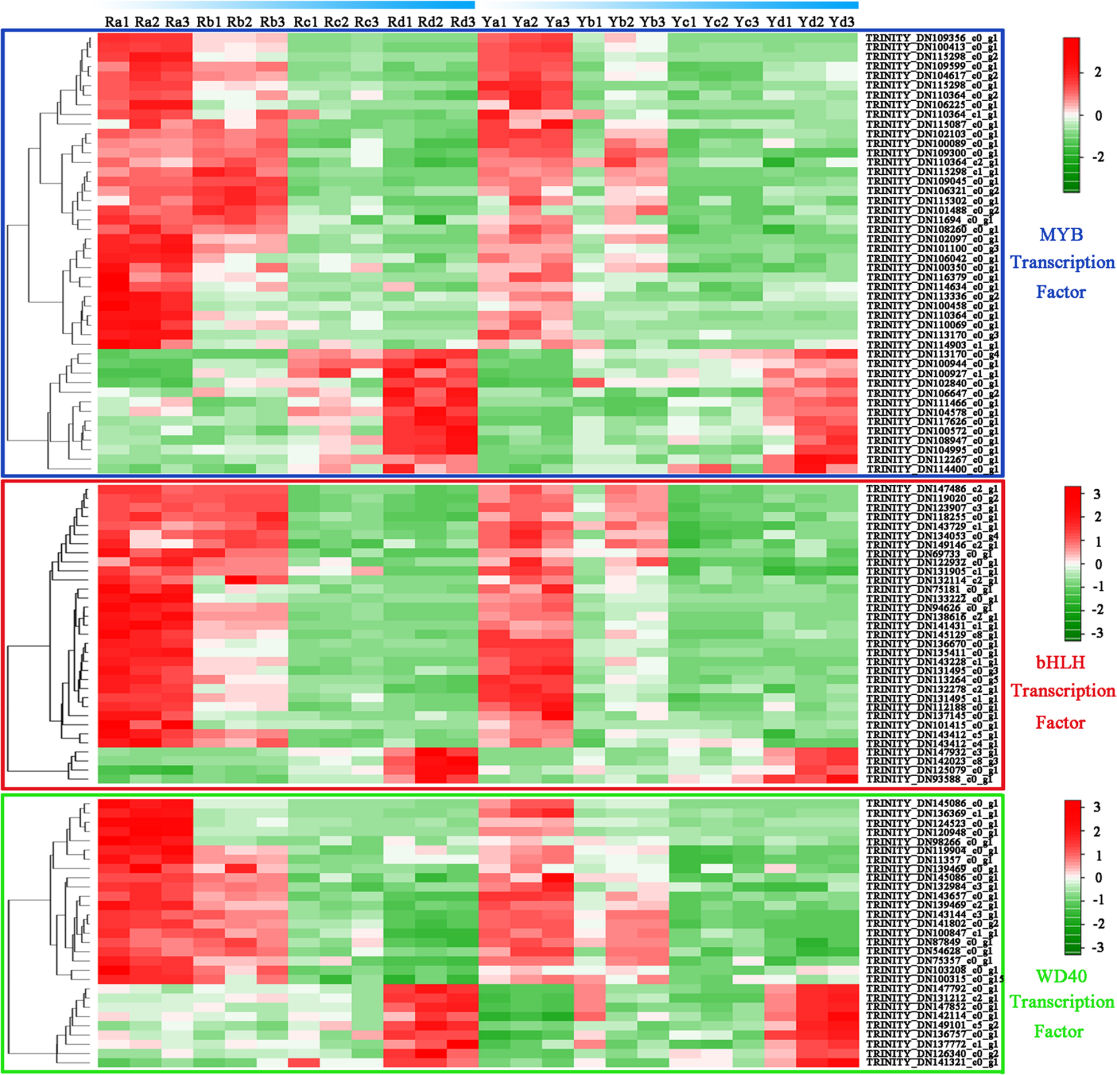
Heat map constructed of MYB, bHLH and WD40 transcription factor family DEGs related to pigment accumulation

MYB-bHLH-WD40 protein complexes play a crucial role in the synthesis and accumulation of plant anthocyanins [15, 16]. Among the DEGs, 46 MYBs, 33 bHLHs, and 29 WD40s were found to be associated with anthocyanin accumulation. Their expression patterns are illustrated in Fig. 7. The expression trends of the 33 MYBs, 29 bHLHs, and 20 WD40s genes were consistent with that of anthocyanin accumulation, meaning the gene transcript abundance decreased with anthocyanin content. The suggestion is that these transcription factors may participate in the positive regulation of anthocyanin biosynthesis. Thirteen MYBs, four bHLHs, and nine WD40s gene expression changes were observed to be contradictory with regard to the anthocyanin content. The suggestion is that these transcription factors may participate in the negative regulation of anthocyanin biosynthesis.

### Construction of co-expressed gene networks

**Fig. 8:**
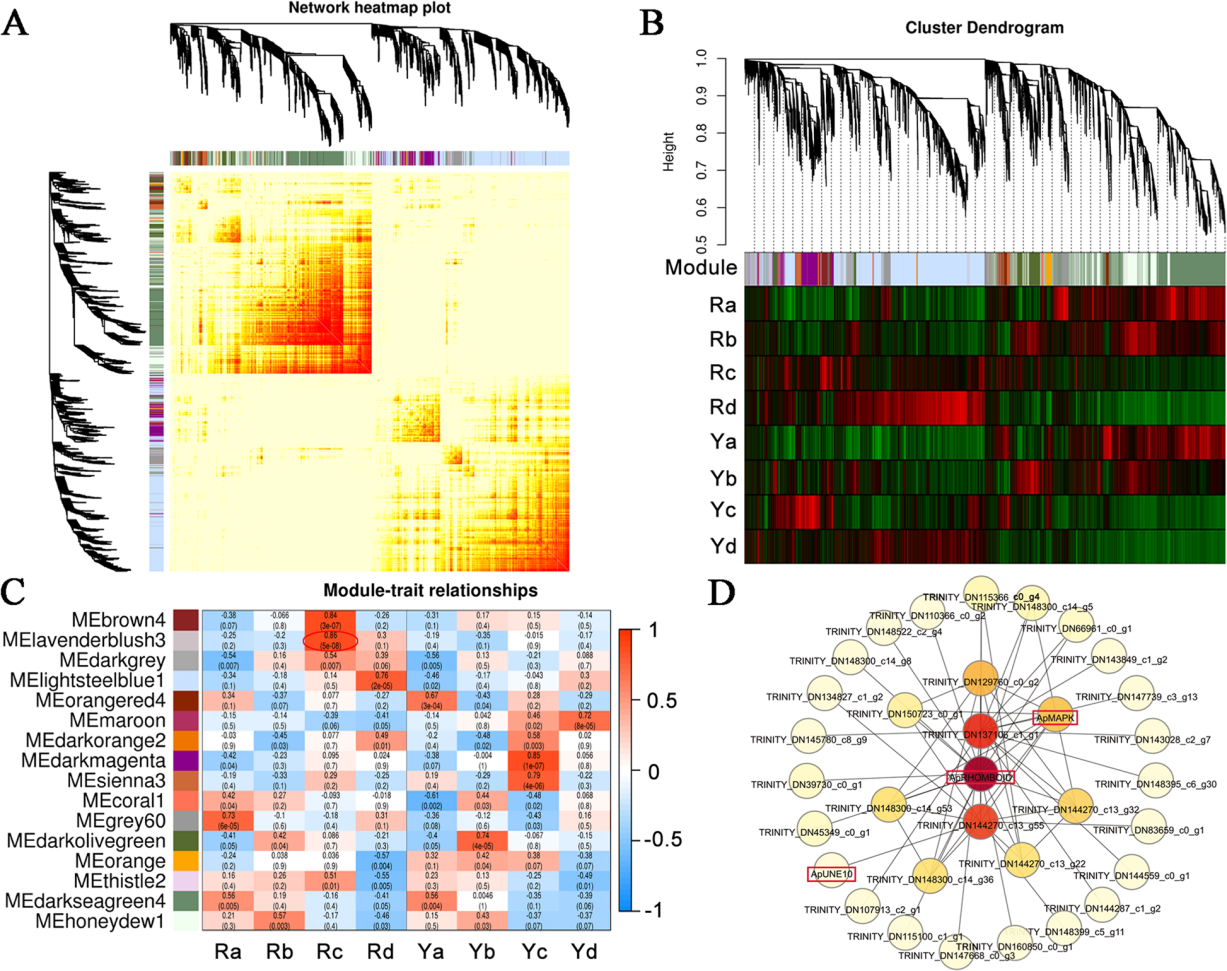
Identification of co-expression network modules via WGCNA analysis in *A. palmatum*. **A** Cluster dendrogram and network heatmap of genes subjected to the co-expression module calculation. **B** Hierarchical clustering and heat map of DEGs presenting 16 modules. **C** Module-trait associations based on Pearson correlations in *A. palmatum* with different leaf colors. The color code from blue to red represents *r*^*2*^ values ranging from − 1 to 1. **D** Gene network of the lavenderblush3 module. The red box represented candidate genes associated with leaf color changes

To investigate the correlation between the two genotypes of *A. palmatum*, WGCNA analysis was performed using transcriptome data. Using the DEGs data and FPKM values, 16 distinct gene modules were defined based on the co-expression patterns throughout the DEGs. These gene modules were distinguished by distinct colors and are presented as a cluster dendrogram and network heatmap (Fig. 8A). The hierarchical clustering of modules and the heat map of DEGs used in the WGCNA analysis are presented in Fig. 8B. In all 16 co-expressed gene networks, the eigengenes of the lavenderblush3 module demonstrated a significant correlation with leaf color. This module contained 1296 genes and possessed a significant association with a correlation coefficient (*r*^*2*^) of 0.86 (Fig. 8C). To further identify the key candidate genes in the lavenderblush3 module, 33 genes were involved in constructing the gene network. Combined with the functional annotation information of unigenes, three genes (*ApRHOMBOID*, *ApMAPK*, and *ApUNE10*) were identified as candidate genes that are associated with leaf color changes (Fig. 8D). In addition to the genes predicted as key candidates, other hub genes in the co-expression modules were identified and are listed in Table S3.

## Discussion

Colorful plants are widely appreciated for the interest they add to gardens and streetscapes. *A. palmatum* is an important ornamental leaf tree that is highly popular worldwide. Differences in pigment content and composition profoundly impact the appearance of color in the leaves [27, 28]. However, the understanding of the precise mechanisms responsible for leaf color change is limited and requires further exploration. In this study, two *A. palmatum* cultivars, a red-leaf cultivar ‘Jinling Danfeng’ and yellow-leaf cultivar ‘Jinling Huangfeng,’ were used to identify the variations in DEGs using transcriptomic analysis. The molecular mechanisms associated with leaf color change due to pigmentation were elucidated through physicochemical analysis and transcriptome experiments. Our findings suggest that the appearance of red and yellow leaves results from a combination of multiple pigments and is closely related to the presence and abundance of various anthocyanins. Based on transcriptomic and WGCNA analyses, the DEGs related to the key genes and transcription factors in the anthocyanin synthesis pathway were prominently activated.

### Involvement of pigment changes in leaf coloration

In an effort to explain the red and yellow coloring of *A. palmatum* leaves, the contents of intermediates in three metabolic pathways were measured and calculated in leaves with different colors. Chlorophyll is a vital substance and is used to facilitate photosynthesis in plants. It is also the main factor contributing to the green coloring of plants [29]. Chlorophyll content in colored leaves tends to be significantly lower than that in green leaves [28]. The chlorophyll a + b content in the red leaves of ‘Jinling Danfeng’ were slightly higher than that of the yellow leaves belonging to the ‘Jinling Huangfeng’ cultivar (Fig. 1D). This suggests that changes in the chlorophyll content may also play a role in differentiating the red and yellow leaves of *A. palmatum*. However, on June 24, the chlorophyll a + b content in ‘Jinling Huangfeng’ leaves were significantly higher than that in the ‘Jinling Danfeng’ leaves. During this period, the golden yellow mature leaves of ‘Jinling Huangfeng’ began to turn green, resulting in a yellow-green color. This suggests that an increase in chlorophyll content was one of the dominant factors affecting yellow-green leaves.

Carotenoids are another important class of pigment. Carotenoids accumulate in tissues and result in the appearance of characteristic colors ranging from yellow to red [30]. The carotenoid content in the red leaves of ‘Jinling Danfeng’ was slightly higher than that in the yellow leaves of ‘Jinling Huangfeng’; however, the difference was not significant (Fig. 1E). This suggests that carotenoids may not be the main factor responsible for the different colors of *A. palmatum* leaves. The type and content of anthocyanins are decisive factors affecting the color of plant tissues [31]. In *A. palmatum* leaves, cyanidin and delphinidin were detected, and their content was significantly higher in red leaves compared to the yellow leaves. As the red color gradually became lighter, the contents of cyanidin and delphinidin also gradually decreased (Fig. 1B, C). These results revealed that cyanidin and delphinidin play an integral role in the color of *A. palmatum* leaves.

### Anthocyanin biosynthesis pathway genes affected leaf color

Enzymes and genes participating in the anthocyanin biosynthesis pathway play an important role in the coloring of colored-leaf plants [32–35]. Several candidate genes involved in pigment formation have been identified in *A. palmatum*. The main structural genes in the anthocyanin biosynthesis pathway are important components of the flavonoid biosynthesis pathway [7, 13, 14]. In this study, KEGG pathways were significantly enriched in anthocyanin biosynthesis during leaf color changes (Fig. 4). KEGG analyses revealed that the gene expression levels of the two genotypes varied between the red and yellow leaves. CHS is the key enzyme responsible for the first committed step of the anthocyanin biosynthesis pathway in plants and was first cloned from *Petroselinum hortense* [36]. *CHS* exists as a multigene family, present in different numbers in different plants, and numerous studies have established that *CHS* can regulate the spatiotemporal accumulation of anthocyanins [32, 37]. *CHI* is the second key gene in plant anthocyanin biosynthesis and catalyzes the isomerization of chalcones. The CHI protein exhibits temporal and spatial specificity, and its expression level has been found to have an important impact on anthocyanin content in plants [34]. As an intermediate hub in anthocyanin biosynthesis, the F3H protein catalyzes the C3 hydroxylation of flavanones to dihydroflavonols. The expression and intensity of F3H is a critical factor in anthocyanin synthesis in various plants [35]. *F3′H* and *F3′5′H* are key genes involved in the synthesis of cyanidin and delphinidin, respectively. Both *F3′H* and *F3′5′H* are cytochrome P450 (CYP450) family genes and determine the hydroxylation pattern of the B-ring [33, 38]. Significant differences were observed in the substrate specificity for the *DFR* gene product. Differential expression of *DFR* and its substrate specificity leads to color variation [39]. *ANS* is a critical regulatory gene that determines the transition of anthocyanins from colorless to colored [40]. The *UFGT* gene was found to promote the conversion of unstable anthocyanins to stable anthocyanins via glycosylation [41]. In the present study, alterations in the transcript levels of anthocyanin biosynthesis structural genes corresponded to changes in cyanidin and delphinidin accumulation. This result is consistent with that of many previous reports. Notably, Ra and Ya had the highest expression of these genes and the most abundant anthocyanin accumulation in the two genotypes of *A. palmatum* (Fig. 5). This may be related to the involvement of anthocyanins in shielding photosystem II against high light stress in young leaves [42, 43].

### Transcriptional regulatory pathways involved in anthocyanins accumulation

Various transcription factors specifically and cooperatively regulate the synthesis, transport, and accumulation of anthocyanins. MYB, bHLH, and WD40 have been indicated as being prominently involved in anthocyanin accumulation by regulating the transcription of structural genes involved in anthocyanin biosynthesis [18, 44, 45]. *ZmC1*, the first MYB transcription factor that regulates anthocyanin biosynthesis in plants, was isolated from *Zea mays* [46]. *CmMYB21* suppresses anthocyanin biosynthesis by directly repressing *CmDFR* expression [45]. In this study, 46 MYBs were found to be significantly associated with anthocyanin accumulation, indicating that MYB plays a crucial role in the regulation of anthocyanin biosynthesis. The bHLH transcription factor is involved in the anthocyanin synthesis pathway via an interaction with the MYBs [47, 48]. It is known to regulate the structural genes (especially *DFR* and *ANS*) involved in anthocyanin biosynthesis. For instance, in *Salvia miltiorrhiza*, SmbHLH60 is a negative regulator of anthocyanin biosynthesis that competes with SmMYC2 in an antagonistic manner and transcriptionally represses *SmDFR* expression [44]. Our data suggested that 33 bHLHs were involved in anthocyanin accumulation. Further research on these genes will help improve the understanding of anthocyanin biosynthesis and leaf color changes in *A. palmatum*. Stable expression of the WD40 transcription factor is also an integral part of anthocyanin accumulation [49]. As a constituent member of the MBW complex, *FhTTG1* (a WD40 gene) can activate anthocyanin biosynthesis-related gene promoters and control anthocyanin accumulation in *Freesia hybrida* [18]. In the present study, transcriptome analysis revealed that 29 WD40s were synchronously expressed with the accumulation of anthocyanins (Fig. 7). These results help to shed light on the establishment of anthocyanin regulatory systems in *A. palmatum*.

### WGCNA of the gene network regulation of the leaf color mechanism

Leaf color is the result of a combination of complex pigments that are regulated by multiple genes. A single gene is often insufficient for leaf color formation. Complex metabolic pathways are usually coordinated by multiple genes [50]. WGCNA networks were constructed, and candidate genes were identified in *A. palmatum*. Sixteen gene expression modules were divided using WGCNA, of which the lavenderblush3 module correlated significantly with leaf color (Fig. 8). *ApRHOMBOID*, *ApMAPK*, and *ApUNE10* were screened as candidate genes in the lavenderblush3 module using WGCNA. These three genes are generally associated with plant growth and development, senescence, signaling, and light response [51–53]. Notably, none of the three genes have been reported to be directly related to anthocyanin and other pigment synthesis pathways. These results indicate that complex metabolic pathways, such as anthocyanin accumulation, require the coordinated expression of gene networks. The lower statistical power of the analysis due to the small sample size is a limitation of our study. The co-expression networks and these candidate genes will help us to further elucidate the coordinated mechanisms of multiple genes involved in the leaf color of *A. palmatum*.

## Conclusion

In conclusion, the transcriptome profiles of two *A. palmatum* genotypes were used to investigate the gene networks controlling the regulation of leaf coloration from spring to summer. The coloration mechanism was revealed in *A. palmatum* by relating the anthocyanin content to the results of RNA-seq analysis. Key regulatory genes that determine leaf color changes were screened and analyzed. Our results revealed that differences in anthocyanin accumulation, particularly the synthesis of cyanidin and delphinidin, led to variations in leaf coloration. The DEGs involved in ‘anthocyanin biosynthesis’ and ‘flavonoid biosynthesis’ were identified by KEGG enrichment analysis. In addition, several differentially expressed structural genes and transcription factors (MYBs, bHLHs, and WD40s) involved in anthocyanin biosynthesis were identified. We selected a module significantly associated with leaf color formation and screened three candidate genes via WGCNA analysis. Our research provides novel insights into the coloration of leaves of *A. palmatum*. In future work, we will focus on the mechanism by which these candidate genes and transcription factors participate in leaf color regulation in *A. palmatum*.

## Methods

### Plant materials and conditions

Two *A. palmatum* genotypes, ‘Jinling Huangfeng’ and ‘Jinling Danfeng,’ were grown at the Acer Research Center, Jiangsu Academy of Agricultural Sciences, China (118.87°E, 32.04°N). Seedlings generated by tissue culture were transplanted into 15 cm pots, grown for one year, and subsequently planted in fields and grown for five years. The fully expanded leaves were harvested every 25 days for construction of the sequencing library and to analyze the content of leaf pigments. At each time point, 10 leaves were considered as one biological sample, and three independent biological replicates (from three plants) were collected from ‘Jinling Huangfeng’ and ‘Jinling Danfeng.’ All samples were immediately placed in liquid nitrogen and stored at − 80 °C until further use.

### Leaf pigment extraction and analysis

Leaf samples (0.2 g) were ground to a fine powder in liquid nitrogen and then homogenized in a 10 ml solvent mixture [methanol, distilled water, methane acid, and trifluoroacetic acid (70:27:2:1, v/v/v/v)] under ultrasonication for 30 min, then transferred to 4 °C for 24 h, and vortexed every 6 h. The samples were centrifuged at 12,000 rpm for 10 min, and the supernatants were filtered through a 0.22 μm reinforced nylon membrane filter (Shanghai ANPEL, Shanghai, China). Anthocyanins were separated using a high-performance liquid chromatography (HPLC) system (Agilent 1290, Santa Clara, CA, USA), employing the following conditions: the mixture was separated after passing through a C18 column (Water, 1.8 μm, 2.1 mm × 75 mm) with an injection volume of 1 µl. The mobile phase comprised a combination of 0.1% formic acid (A) and chromatographic methanol (B) at a flow rate of 0.3 ml/min. From 0 to 7 min, the mobile phase was 90–10% A, 10–90% B, rising to 10–90% A, 90–10% B by 10 min. A triple quadrupole LC-MS/MS system (AB SCIEX QTRAP 6500 LC-MS/MS; Framingham, MA, USA) was used for detection and quantification of the anthocyanins. Six authentic standards (pelargonidin, cyanidin, peonidin, delphinidin, petunidin, and malvidin) were used for the qualitative and quantitative analyses.

The acetone extraction method was used to determine the chlorophyll and carotenoid contents [28]. Fresh leaf tissue (0.1 g) was chopped and immersed in 10 ml of a 9:1 (v/v) mixture of acetone: 0.1 M NH_4_OH and chlorophyll a, chlorophyll b, and carotenoids were extracted. Three biological replicates with technical duplicates were measured for each sample at each collection time point.

### RNA extraction and transcriptome sequencing

Leaves from four different periods of ‘Jinling Huangfeng’ and ‘Jinling Danfeng’ were collected for RNA extraction and cDNA library construction. Three biological replicates were performed for each material. Total RNA was isolated from the leaves using the RNAiso reagent (TaKaRa, Tokyo, Japan). The concentration and purity of the RNA samples were subsequently measured using a NanoPhotometer 2000 spectrophotometer (IMPLEN, CA, USA), a Qubit 3.0 Fluorometer (Life Technologies, CA, USA), and an Agilent Bioanalyzer 2100 system (Agilent Technologies, CA, USA).

Library construction was performed using the NEBNext® Ultra™ RNA Library Prep Kit for Illumina® (NEB, USA). Magnetic beads linked with oligo (dT) beads were used to enrich mRNA from the highly purified total RNA. Fragmentation buffer was added to the enriched mRNA to break it into shorter fragments, which were reverse transcribed to synthesize cDNA. PCR amplification was used to enrich the cDNA. The PCR products were purified with AMPure XP beads, and strand-specific library construction was completed. Qualified libraries were sequenced on an Illumina HiSeq Xten platform (Illumina Inc., USA), and 150 bp paired-end reads were generated.

### Data processing

High-quality reads were subjected to strict quality control for subsequent data analysis. To obtain high-quality reads, the adapters present, sequencing primers and low-quality reads were removed from the original data. Effective rate, quality score (Q20 and Q30) and GC content were strictly checked and controlled. After obtaining high-quality cleaned data, sequence assembly was performed using Trinity software [54]. The FPKM (Fragments Per Kilobase of transcript per Million mapped reads) value of each gene for each sample was used to calculate gene expression. DESeq R package was designated as the differential expression analysis between sample groups [55]. During the detection of differentially expressed genes, |log2 (Fold Change)| ≥1 and FDR (False Discovery Rate) < 0.01 were used as screening criteria.

### Functional gene annotation and DEGs enrichment analysis

Unigene sequence functions were annotated in seven databases using BLAST software (v2.2.31, http://blast.ncbi.nlm.nih.gov/Blast.cgi) [56], including: Nr (non-redundant protein sequences, www.ncbi.nlm.nih.gov/refseq) database, Nt (non-redundant nucleotide sequences, www.ncbi.nlm.nih.gov/nuccore) database, KOG/COG (Eukaryotic Ortholog Groups, www.ncbi.nlm.nih.gov/COG/) database, GO (Gene Ontology) database, http://www.geneontology.org/page/go-database), Pfam (Homologous Protein family, http://pfam.xfam.org/) database, Swiss-Prot (a manually annotated and reviewed protein sequence database, www.uniprot.org) database and KEGG database (www.kegg.jp/kegg/kegg1.html) [57–59]. GOseq (v1.48.0), topGO (v2.48.0) and KOBAS (v2.0) software with default parameters were used to test the enrichment analysis of the DEGs in GO and KEGG pathways, respectively [60–62]. Q value was applied to select the significant terms.

### WGCNA analysis

The WGCNA package (R package v1.70-3 with default version) with default parameters was used to construct the gene co-expression network [63, 64]. The normalized values (FPKM values) of all the genes were inputted to WGCNA pipeline. Based on the gene expression correlation, a soft threshold (power value = 11, scale free topology fitting index R^2 > 0.9) was selected to construct an adjacency matrix. Genes involved in WGCNA were divided into several modules, and genes with identical expression patterns were grouped into the same module. Genes that were not accepted for co-expression analysis were classified as gray modules. The WGCNA network, module division results, and sample trait joint analysis of the correlation coefficient between modules and samples were combined. Cytoscape software was used to visually export the genes from each module.

### Quantitative real-time PCR (qRT-PCR)

The DEGs expression profiles were validated via qRT-PCR analyses. The same biological RNA samples (number of independent biological samples and replicates) were used for sequencing and qRT-PCR experiment. The qRT-PCR protocol and techniques used have been previously described by Zhu et al. (2020) [65]. The *ApActin* gene (accession no.: MN026864) was used as a quantitative control for *A. palmatum* [65]. The specific primers used are listed in Table S1. The relative expression values were calculated using the 2^–ΔΔCt^ algorithm [66]. Three biological replicates were used for the qRT-PCR analysis.

## Supplementary Information

**Additional file 1.**

**Additional file 2.**

**Additional file 3.**

**Additional file 4.**

**Additional file 5**.

**Additional file 6.**

**Additional file 7.**

**Additional file 8.**

## Data Availability

The raw data of 24 samples generated in this study have been deposited in the NCBI Short Read Archive (SRA) under the BioProject accession number PRJNA873794.
