# Supplementary figures and images for: Transcriptome profiling provides insights into leaf color changes in two Acer palmatum genotypes
Source: BMC Plant Biol. 2022 Dec 16;22:589. doi: 10.1186/s12870-022-03979-x (PMC9756493; doi:10.1186/s12870-022-03979-x)

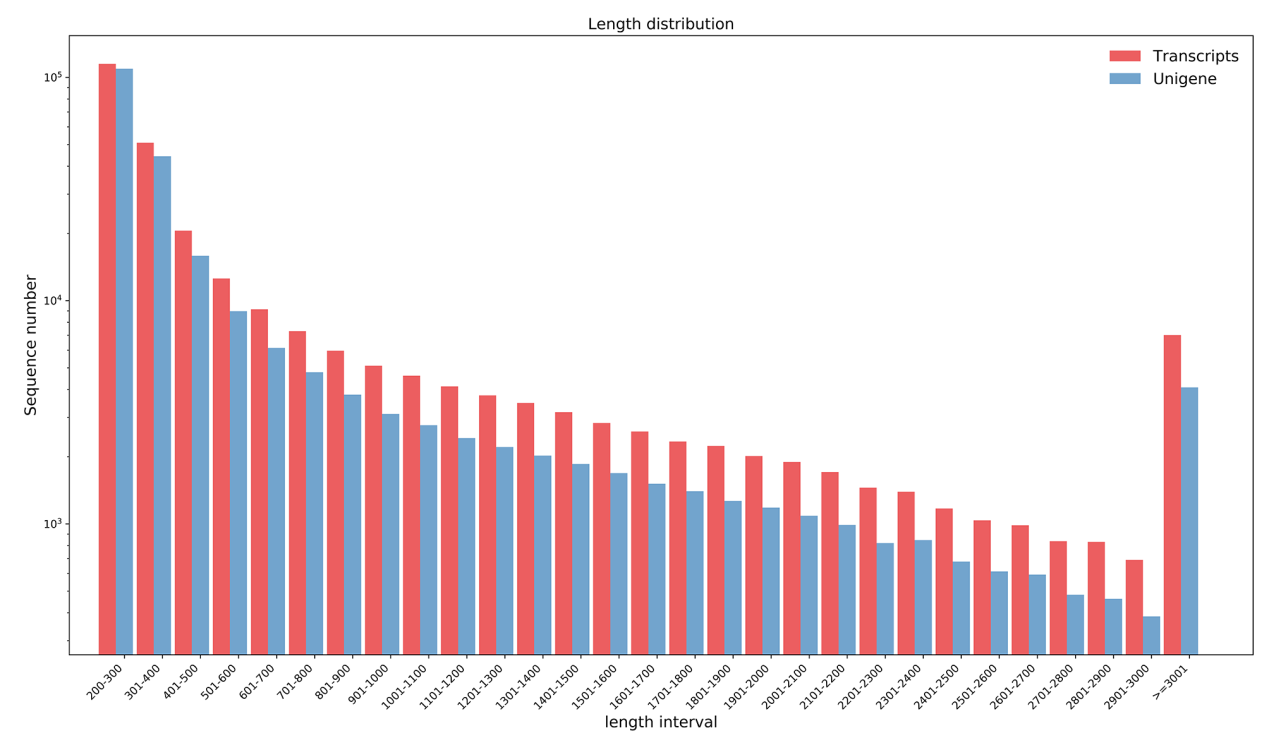


**Fig. S1** Sequence length distribution map of assembly results.

Supplement: Supplementary file 1 — Additional file 1. [file 12870_2022_3979_MOESM1_ESM.docx]

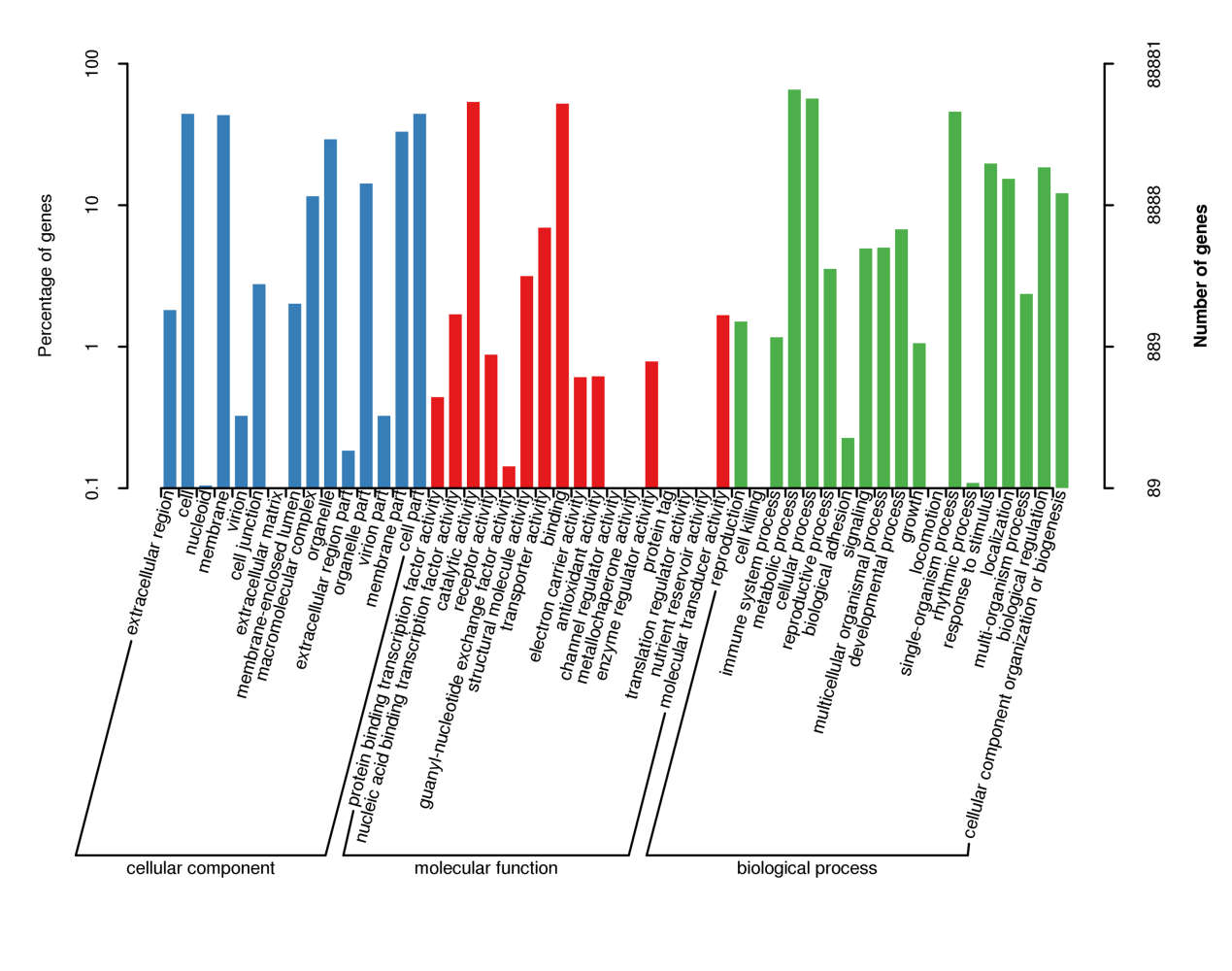


**Fig. S2** GO classification of all unigenes.

Supplement: Supplementary file 2 — Additional file 2. [file 12870_2022_3979_MOESM2_ESM.docx]

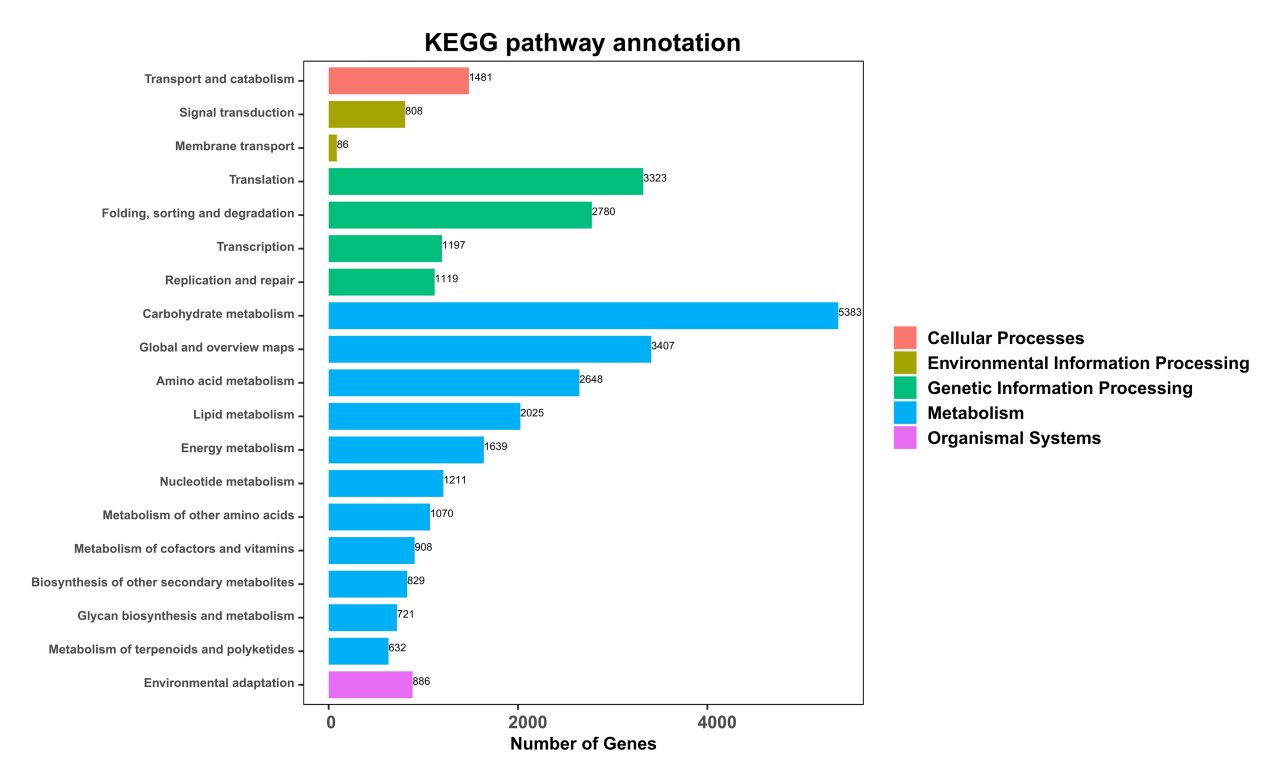


**Fig. S3** KEGG pathway annotation of all unigenes.

Supplement: Supplementary file 3 — Additional file 3. [file 12870_2022_3979_MOESM3_ESM.docx]

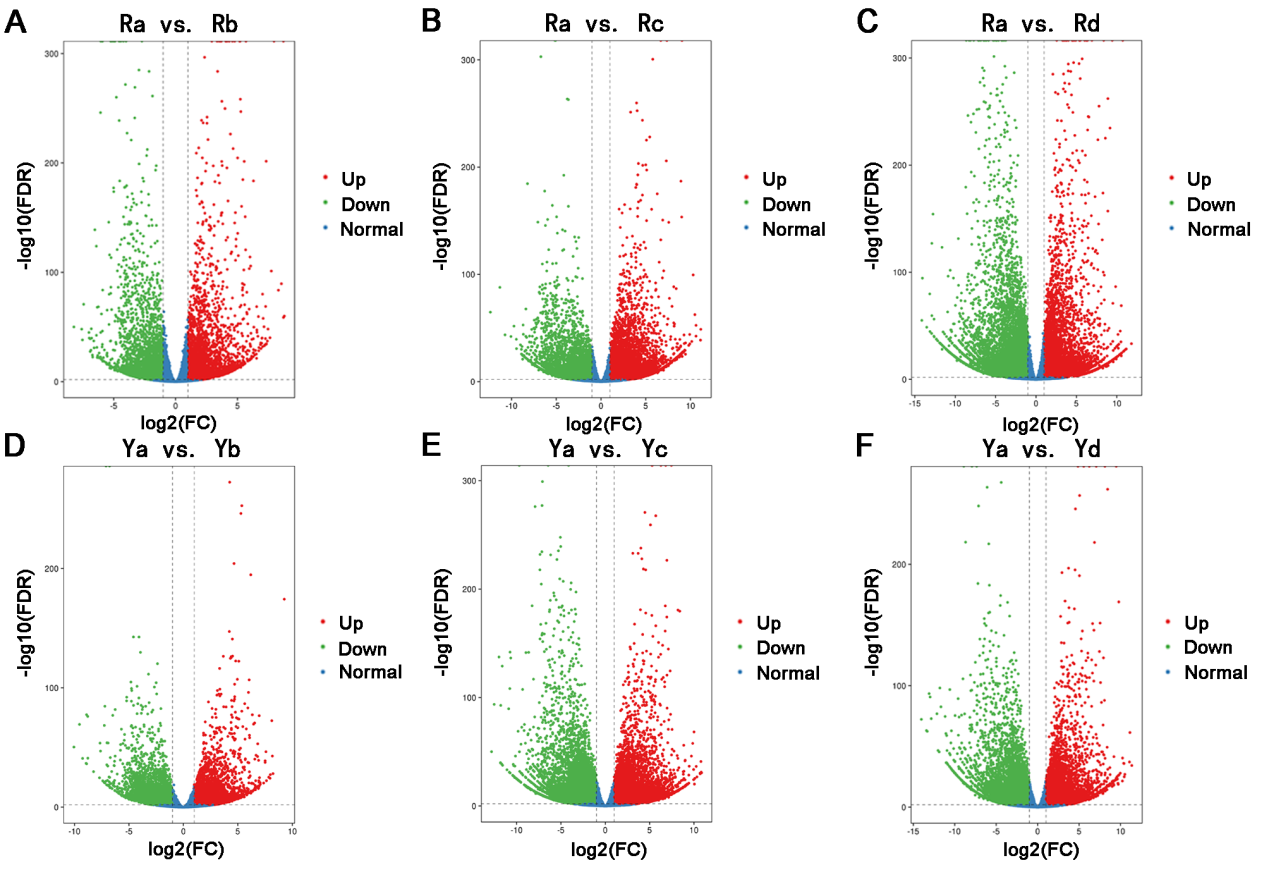


**Fig. S4** Differences in the abundance of genes. (**A**) Ra vs. Rb; (**B**) Ra vs. Rc; (**C**) Ra vs. Rd; (**D**) Ya vs. Yb; (**E**) Ya vs. Yc; (**F**) Ya vs. Yd.

Supplement: Supplementary file 4 — Additional file 4. [file 12870_2022_3979_MOESM4_ESM.docx]
