# Supplementary material for: Transcriptome profiling provides insights into leaf color changes in two Acer palmatum genotypes
Source: BMC Plant Biol. 2022 Dec 16;22:589. doi: 10.1186/s12870-022-03979-x (PMC9756493; doi:10.1186/s12870-022-03979-x)
Supplement: Supplementary file 5 — Additional file 5. [file 12870_2022_3979_MOESM5_ESM.doc]

**Table S1. Primer sequences used in this study.**

| Primer | Sequences (5'-3') |
| --- | --- |
| ApCHS-F | TGGGAATCTCAGACTGGAACT |
| ApCHS-R | TGGCTCTCATTTTCTCAGGTT |
| ApCHI-F | TGGCTTCTTCACTGCCTGTAG |
| ApCHI-R | AAGTGCCTGTGACCTCTTCTC |
| ApF3H-F | ATTCCCTGGTCTAAACATCTG |
| ApF3H-R | ATTCTATCTGCTTTCCTGCC |
| ApF3'H-F | TACAGATTTCAACTATCAACCA |
| ApF3'H-R | CTAAACAAGCCTTTTTTCGTG |
| ApDFR-F | TCTCACTCAGCCACCATTCT |
| ApDFR-R | AAATCCCAAATCCAAAATCT |
| ApANS-F | GTTACTGCCAAATGTGTTCCA |
| ApANS-R | GACAAGTCCCCTGTGAAGAAT |
| ApUFGT-F | TGGCAGTGTTGGTTGCTATG |
| ApUFGT-R | ATAACAGTTTTCACAGCGTCC |
| ApActin-F | CACTTTGGATGATGGATCTCTAC |
| ApActin-R | TCTCTTCCTGTCCTTACGCTC |
