# Supplementary material for: Transcriptome profiling provides insights into leaf color changes in two Acer palmatum genotypes
Source: BMC Plant Biol. 2022 Dec 16;22:589. doi: 10.1186/s12870-022-03979-x (PMC9756493; doi:10.1186/s12870-022-03979-x)
Supplement: Supplementary file 6 — Additional file 6. [file 12870_2022_3979_MOESM6_ESM.docx]

**Table S2.** Summary of the transcriptome sequencing data and quality control information.

| Sample | Raw Reads | Clean Reads | Clean bases | Effective Rate (%) | Q20 (%) | Q30 (%) | GC (%) |
| --- | --- | --- | --- | --- | --- | --- | --- |
| Ra1 | 63336886 | 61747838 | 9.26 G | 97.49 | 97.45 | 93.45 | 43.99 |
| Ra2 | 56506484 | 55037738 | 8.26 G | 97.40 | 97.67 | 93.93 | 43.86 |
| Ra3 | 59376532 | 57682708 | 8.65 G | 97.15 | 97.38 | 93.31 | 43.80 |
| Rb1 | 40755438 | 40012842 | 6.00 G | 98.18 | 96.44 | 90.74 | 43.77 |
| Rb2 | 47603982 | 46407416 | 6.96 G | 97.49 | 96.55 | 90.95 | 43.56 |
| Rb3 | 52861060 | 51850550 | 7.78 G | 98.09 | 95.82 | 89.19 | 43.60 |
| Rc1 | 55876658 | 53832460 | 8.07 G | 96.34 | 97.34 | 92.52 | 43.83 |
| Rc2 | 50034920 | 48624852 | 7.29 G | 97.18 | 97.18 | 92.38 | 43.90 |
| Rc3 | 52710212 | 51725900 | 7.76 G | 98.13 | 96.51 | 90.54 | 44.24 |
| Rd1 | 52938002 | 51397964 | 7.71 G | 97.09 | 96.79 | 91.18 | 43.91 |
| Rd2 | 49473904 | 47996488 | 7.20 G | 97.01 | 97.43 | 92.95 | 43.61 |
| Rd3 | 48363346 | 46850754 | 7.03 G | 96.87 | 97.17 | 92.38 | 43.63 |
| Ya1 | 52271072 | 51216596 | 7.68 G | 97.98 | 96.70 | 90.96 | 44.29 |
| Ya2 | 55301650 | 54102988 | 8.12 G | 97.83 | 97.50 | 93.33 | 43.75 |
| Ya3 | 54591412 | 53608374 | 8.04 G | 98.20 | 96.64 | 91.14 | 43.91 |
| Yb1 | 54841166 | 53864734 | 8.08 G | 98.22 | 96.64 | 91.13 | 43.66 |
| Yb2 | 52574944 | 51722552 | 7.76 G | 98.38 | 96.57 | 90.70 | 43.84 |
| Yb3 | 48089990 | 47013216 | 7.05 G | 97.76 | 96.94 | 92.01 | 43.81 |
| Yc1 | 46423766 | 44770084 | 6.72 G | 96.44 | 96.36 | 90.59 | 44.36 |
| Yc2 | 49135164 | 47463522 | 7.12 G | 96.60 | 96.90 | 91.71 | 43.93 |
| Yc3 | 49623480 | 48377350 | 7.26 G | 97.49 | 96.49 | 90.81 | 44.01 |
| Yd1 | 52503702 | 50448174 | 7.57 G | 96.08 | 96.67 | 91.05 | 44.85 |
| Yd2 | 62730594 | 60911356 | 9.14 G | 97.10 | 97.51 | 93.53 | 43.97 |
| Yd3 | 57943306 | 56427956 | 8.46 G | 97.38 | 97.40 | 93.27 | 43.66 |
